# Tantrums, Emotion Reactions and Their EEG Correlates in Childhood Benign Rolandic Epilepsy vs. Complex Partial Seizures: Exploratory Observations

**DOI:** 10.3389/fnbeh.2018.00040

**Published:** 2018-03-09

**Authors:** Michael Potegal, Elena H. Drewel, John T. MacDonald

**Affiliations:** ^1^Program in Occupational Therapy, Center for Allied Health Professions, University of Minnesota, Minneapolis, MN, United States; ^2^Department of Neuro and Behavioral Psychology, St. Luke’s Children’s Center for Autism and Neurodevelopmental Disabilities, Boise, ID, United States; ^3^Department of Neurology, Medical School, University of Minnesota, Minneapolis, MN, United States

**Keywords:** anger, anxiety, fear, hemispheric laterality, interictal epileptiform discharges, sadness

## Abstract

We explored associations between EEG pathophysiology and emotional/behavioral (E/B) problems of children with two types of epilepsy using standard parent questionnaires and two new indicators: tantrums recorded by parents at home and brief, emotion-eliciting situations in the laboratory. Children with Benign Rolandic epilepsy (BRE, *N* = 6) reportedly had shorter, more angry tantrums from which they recovered quickly. Children with Complex Partial Seizures (CPS, *N* = 13) had longer, sadder tantrums often followed by bad moods. More generally, BRE correlated with anger and aggression; CPS with sadness and withdrawal. Scores of a composite group of siblings (*N* = 11) were generally intermediate between the BRE and CPS groups. Across all children, high voltage theta and/or interictal epileptiform discharges (IEDs) correlated with negative emotional reactions. Such EEG abnormalities in left hemisphere correlated with greater social fear, right hemisphere EEG abnormalities with greater anger. Right hemisphere localization in CPS was also associated with parent-reported problems at home. If epilepsy alters neural circuitry thereby increasing negative emotions, additional assessment of anti-epileptic drug treatment of epilepsy-related E/B problems would be warranted.

## Introduction

Pediatric epilepsy is among the most common neurological diseases of childhood. Besides cognitive deficits, pediatric epilepsy is associated with significant emotional/behavioral (E/B) problems, some of them occurring more frequently than they do in other serious childhood diseases, such as asthma, diabetes or cardiac conditions (Hoare, 1984; Rodenburg et al., 2005). These problems often include “internalizing” anxiety and depression, but “externalizing” inattention, hyperactivity, anger and aggression are also reported. Seizure factors known to contribute to E/B risk include earlier onset, multiple types and higher frequency (Austin and Caplan, 2007). Nevertheless, we still don’t know why only some children experience E/B problems. This uncertainty can be distressing to parents and puzzling to physicians (Smith et al., 2007), so E/B problems associated with epilepsy may be under-diagnosed and under-treated (Hanssen-Bauer et al., 2007; Verrotti et al., 2014). Although some anti-epileptic drugs (AEDs) appear useful in treating E/B problems (Pressler et al., 2005), physicians are unlikely to use them to treat such problems until a clear and direct connection is made between children’s psychopathology and their epilepsy.

Some systematic associations between epilepsy type and particular E/B symptoms have been reported. Two studies of 5–16 years old with complex partial seizures (CPS) found greater internalizing, but not externalizing problems on the Child Behavior Checklist (CBCL, Schoenfeld et al., 1999; Caplan et al., 2004). Conversely, executive dysfunction and impulsivity are more characteristic of frontal than temporal lobe epilepsy (Culhane-Shelburne et al., 2002). In two of six diagnostically more specific studies, children with Benign Rolandic Epilepsy (BRE) or “central-temporal spikes” were rated by parents as higher than controls on “distractibility, concentration and impulsiveness” with the most significant difference being in “temper” (on laboratory-specific Swedish questionnaires, Croona et al., 1999) or as having significantly higher CBCL subscale scores including Social Problems and Delinquent Behavior (in German translation, Weglage et al., 1997). In contrast to these whole-group comparisons, the other four studies identified subgroups of children with BRE, varying from 7.6% (Tovia et al., 2011) through about 30% (Yung et al., 2000; Massa et al., 2001) to 50%–64% (Saeed et al., 2014) of their respective samples, who had behavioral problems (inattention/distractability, hyperactivity, oppositional and/or aggressive behavior) based on one or another mix of parent and teacher checklists, neuropsychological or school psychology evaluations, and formal psychiatric diagnoses. However, these studies used different methodologies that are difficult to compare; a direct comparison of CPS vs. BRE behavioral profiles using the same methods would be most informative.

Results of the BRE studies underscore the importance of identifying and characterizing subgroups with E/B problems. Indeed, the affected subgroup in Massa et al. (2001) was differentiated by six pathophysiological features including intermittent slow-waves, multiple asynchronous spike-wave foci, and many interictal abnormalities during wakefulness and sleep. These results agree with findings that behavior problems are associated with interictal epileptiform discharges (IEDs), e.g., sharp waves, spikes and spike-wave complexes (Pressler et al., 2005). Important as these results are, they were obtained through extended (and sometimes repeated) 2–24 h recordings and labor-intensive reading/counting of EEGs. It would be more clinically useful to have a simplified system for relating children’s behaviors to abnormal waveforms routinely scrutinized in an office EEG.

Another source of E/B variability is the laterality of seizure foci. In adults, a preponderance of studies implicate left hemisphere foci in psychiatric problems, typically anxiety (Smith et al., 2007). Similar results, albeit mixed and weak, are reported in older studies of children’s inattention and internalizing problems. More recently, 6–15 years old with right localized partial epilepsies reportedly showed more emotional impairments (anger, disruptive behavior) than those with left hemisphere foci (Mathiak et al., 2009). Our related observations are noted below.

Information sources for E/B function are also at issue. To date, most behavioral measures have been parent (or teacher) report via standard questionnaires or structured interviews. These are retrospective, generalized over a child’s history, and potentially biased by parents’ own issues. Here, we introduce two other sources of information; directly observed emotion responses elicited in episodes from the Laboratory Temperament Assessment Battery (Lab-TAB) and parent’s on-the-spot observations of temper tantrums at home. Lab-TAB episodes are widely used for eliciting children’s emotional reactions to assess temperament and psychopathology (Gagne et al., 2011; Clifford et al., 2015). Akin to the physiological challenges used in clinical EEGs, we used three brief Lab-TAB episodes to elicit mild fearfulness, frustration and disappointment during EEG recording (all episodes did end happily for the child).

Tantrums are an overlooked but important and frequently troublesome aspect of E/B dysregulation that can degrade quality of life for both parent and child. Older studies found increased prevalence of tantrums in children with epilepsy (Keating, 1961; Nuffield, 1961; Elithorn, 1974). Our study was inspired by a pilot survey of 5–12 years old in the University of Minnesota’s Pediatric Neuropsychology Clinic that revealed a remarkable 22 of 23 (96%) of those with epilepsy had tantrums (unpublished observations). Note that tantrums are not normative in this age range; tantrum prevalence estimates for these ages vary from 11%–30% (Potegal, 2018). We, like Mathiak et al. (2009), noted that right hemisphere localization was associated with longer and more severe tantrums.

Frequency and duration are significant tantrum parameters, but tantrum content, what children actually do, is equally important. Our work in other clinical (Potegal et al., 2009) and non-clinical populations (Potegal and Davidson, 2003; Green et al., 2011) shows that tantrums are a mix of anger (indicated by, e.g., stamping, hitting, screaming) and sad distress (indicated by whining, crying, comfort-seeking). Tantrum anger to distress ratios are clinically useful, e.g., identifying child psychiatry inpatients with anxiety diagnoses by the lower anger and greater distress of their on-ward “rages” (Potegal et al., 2009). Similarly, in our Neuropsychology Clinic survey, tantrums of children with CPS had the lowest levels of anger relative to distress, consistent with their “internalizing” symptoms.

Tantrum emotions may have lateralized hemispheric substrates. In a comparison of tantrum prone vs. non-tantrum prone 4 years old, parent reported and Lab-TAB measures of anger were associated with greater EEG activation (lower alpha power) in left temporal lobe whereas sadness was associated with right frontal activation (Potegal and Stemmler, 2010). These finding are in keeping with the now standard model of anger being associated with left hemisphere activation (Harmon-Jones et al., 2010) vs. anxiety and depression being related to right hemisphere activation (Thibodeau et al., 2006; see Andrews et al., 2000 for the corresponding laterality of seizures triggered by anger vs. fear).

The presence of one or another type of epilepsy, the lateralization of seizure foci, and the extent of EEG pathophysiology might account for the occurrence and characteristics of children’s tantrums and/or the variability of their E/B problems. To begin resolving E/B variability, we explored four specific hypotheses about the relationship between emotion reactions in the clinic, tantrums at home, and EEG characteristics observed in 1 h recording sessions of children with BRE or CPS. Family factors were controlled by comparing these children to their unaffected siblings. Our hypotheses were:

1. Children with either BRE and CPS have significantly more frequent and/or longer tantrums at home than siblings. Relative to each other, BRE tantrums express more anger; CPS tantrums express more distress.
2. On parent reports of E/B functioning, children with BRE show more anger, social conflict and externalizing problems; children with CPS show more sadness, social withdrawal and internalizing problems.
3. Children with BRE will show more frequent and/or intense anger in response to emotion challenges; children with CPS will show more sadness. One or both epilepsy groups will show greater emotion responses than sibling controls.
4. EEG localization and/or pathology in the BRE group will correlate with observed and/or parent reported anger intensity and externalizing behaviors; EEG localization and/or pathology in the CPS group will correlate with sadness and/or fear.

## Materials and Methods

### Participants

Thirteen 5–12 years old with diagnoses of CPS, 6 with BRE, and 11 of their siblings (nine from the CPS group, two from the BRE group) in the same age range were recruited from the practices of participating pediatric neurologists (Drs. J. T. MacDonald, S. Rothman and the Minnesota Epilepsy Group). Diagnoses of CPS or BRE were based upon classical clinical criteria and EEG recordings (International League Against Epilepsy, 1989). Exclusion criteria for all groups included: IQ < 70; major speech/language, sensory, or motor impairments, autism, or brain tumor; major medical problems or seizures secondary to other medical treatment.

Parents consenting to a verbal question or letter from their physician were contacted by telephone. Those indicating interest were sent a written consent form; consent was obtained after they had reviewed the form and had all questions answered. Age of seizure onset, seizure types, current seizure frequencies, estimates of lifetime seizures; EEG, MRI and CT results; and medication history were extracted from medical records. Families were compensated $100 for each child who participated. This study was carried out in accordance with the recommendations of the Belmont Report, University of Minnesota Institutional Review Board (IRB) with written informed consent from all subjects. All subjects gave written informed consent in accordance with the Declaration of Helsinki. The protocol was approved by the Faculty Social-Behavioral Committee of the University of Minnesota IRB.

### Measures

#### Tantrum Calendar/Check-In Calls

Parents noted the onset and duration of each tantrum on a 6 week calendar. Weekly check-in calls asking about the most recent tantrum fostered compliance and allowed assessment of calendar reliability. Tantrum frequency and mean duration were calculated from the completed calendars.

#### Post-Tantrum Checklist

A Post-Tantrum Checklist was completed by parents immediately following each of 1–3 tantrums. It assessed the event that triggered the tantrum as well as 14 anger behaviors (e.g., hit, kick, scream) and five distress behaviors (e.g., whine, cry, comfort-seeking) each scored as 0, 1 (one or two occurrences), or 2 (three or more occurrences). Parents also rated overall tantrum severity on a 1–5 scale (3 was “average” severity; 1 and 5 were “among the least severe” in the last month and “among the most severe”, respectively). Choices for the child’s post-tantrum behavior/mood included “returned to his/her usual activities as if nothing happened” and “seemed in a bad mood” (Checklist available from MP).

#### Questionnaires

Parents completed the 6–18 years Child Behavior Checklist (CBCL; Achenbach and Rescorla, 2001) and the Parenting Stress Index (PSI; Abidin, 1990). Socio-economic variables, i.e., parent age, education, family income and number of children in the family were recorded.

#### EEG Recording

Children had a 1 h waking EEG using a 128 channel sensor net (Geodesic EEG System 300, Michel et al., 2004). Electrode impedances were set < 50 KΩ; data were bandpass filtered (0.5–50 Hz) and sampled at 1–200 Hz referenced to vertex. The recording sequence was: 10 min baseline (child sitting still), hyperventilation challenge (<3 min), 10 min baseline, three mild emotion challenges (2–4 min each), final 10 min baseline.

#### Emotion Challenges

Parents were informed beforehand about the challenges and observed them from the control room. They were told that they could terminate testing at will. None did so. At the end of each challenge, parents noted their child’s overall response on a color-coded 1–10 scale that also had facial expression cartoons and icons representing emotion intensity (scales available from MP). Children’s behavior was videotaped.

##### Spiders

This episode first tapped phobic-like responses by asking the child to take a large (toy) spider out of its cage and match it to one of several spider pictures (spiders are the most frequent item on children’s free self-report of scary things, Muris et al., 1997). Social anxiety was then tapped by telling the child that s/he had 2 min to prepare a speech on spiders to “some people waiting down the hall”. After 2 min, s/he was told that the people had left, so no speech was needed.

##### Unsolvable puzzles

The child was asked to assemble three Wechsler Intelligence Scale for Children-III Object Assembly puzzles (switching of pieces made assembly impossible). At the 60 and 90 s points in each 2 min trial they were prompted to “Work a little faster”. After the third puzzle, the experimenter exclaimed “Oh my! These puzzles all have missing pieces”; the child was then given a final, easily solvable puzzle and praised for solving it.

##### Worst prize

Children were told that they had worked so hard that when they were done they would get the prize they had pre-selected from among a set of prizes. Unwrapping the box left by an assistant, they found the least desirable prize, a scribbled on and torn rag doll. After 1 min, the assistant returned and said “That’s not the toy you wanted”. The assistant left again but returned 1 min later having “found” the desired prize, which was given to child. Following completion of all episodes, children reported the intensity of their feelings during each one on 1–10 scales like those given to parents.

### Data Analyses

#### Groups

The behavior scores of the two siblings of children with BRE were not statistical outliers on the score distributions of the nine CPS siblings, so all sibling data were pooled into a single Sib group (*n* = 11).

#### Anger/Distress Index

Relative proportions of anger and distress in each checklisted tantrum was quantified with the Anger/Distress Index (A/D-I). *A/D-I* = *(ΣA − ΣD)****/****(ΣA + ΣD)* where *ΣA* and *ΣD* are the respective sums of anger and distress behaviors. Sample *A/D-I* values: 1.0 = anger only, 0.0 = equal mix, −1.0 = distress only.

#### EEG Pathophysiology

##### Abnormality score

All subjects were awake during recording. Digitally stored EEG was visually scored offline by a blinded pediatric neurologist (JTM). Abnormal EEG patterns were defined as rapid shifts in normal waking background to: (1) higher voltage bursts of rhythmic theta or delta activity; and/or (2) IEDs including spikes and spike-wave complexes (Westmoreland, 1996; Geyer et al., 1999; Koutroumanidis et al., 2004; Michel et al., 2004; Jing et al., 2010). Table 1 quantifies abnormalities: Brief bursts of moderate to high voltage theta were scored as 1; higher voltage theta with sharp or saw-tooth morphology was scored as 2. For IEDs, brief spikes were scored as 1, spike-wave patterns as 2 (Blume et al., 1984; Devinsky et al., 1988; Williamson et al., 1993; Bare et al., 1994). To avoid over-counting abnormal waveforms, the total Abnormality score for each EEG was the sum of the highest scores for each of the three waveforms (range 0–5).

**Table 1:** EEG pathophysiology scoring systems.

	*EEG abnormality scores*		
Score	0	1	2	
Delta	Absent	Present	
Theta	Absent	Present	Higher, sharp or saw-tooth
IED	Absent	Brief Spikes	Spike and wave
		*EEG laterality score*
Left = 1	Left > Right = 2	Left ≈ Right = 3	Left < Right = 4	Right = 5

*IED are interictal epileptiform discharges*.

##### Laterality score

Focal abnormal waveforms involving at least three ipsilateral leads were scored for laterality as in Table 1: 1 (exclusively left), 3 (equally distributed) or 5 (exclusively right). Left or right-weighted scores of 2 or 4 were given when abnormal waveform amplitude in one hemisphere was at least twice that in the other hemisphere (Sharbrough, 1993; Kellaway, 2000; Lee et al., 2000; Foldvary et al., 2001).

Differences between groups were tested with univariate and multivariate analysis of variance (MANOVA) and covariance (MANCOVA). Siblings of children with epilepsy often have EEG abnormalities (Croona et al., 1999; Ottman, 2001; Bali et al., 2007). These children, as well as those with EEGs within normal limits, were included in correlations with abnormality. *Post hoc* linear regression was used to distinguish the contributions of the various EEG abnormalities to behavior scores. Correlations with laterality included only children with some EEG abnormality (regardless of level). We report statistical tests only for correlations with *r* > 0.4 and for which visual inspection of scatter plots showed general trends that were not due to a few outliers.

## Results

### Family Demographics

Family demographics are in Table 2. One CPS family was Asian, another African-American, all others were white. For the CPS group, 55% of fathers and 77% of mothers completed college; for the BRE group, 75% of both mothers and fathers completed college. Families reported a range of incomes; most were middle class. Out of 19 possible family stressors, about 40% of families in both groups reported none, 80%–90% reported ≤ 3.

**Table 2:** Group composition and family demographics.

		CPS	BRE	Sibs
*N*		13	6	11
Mean age (years)		9.2 ± 1.7	9.0 ± 2.3	9.6 ± 2.7
% (number) boys		69 (9)	67 (4)	55 (6)
% (number) on AEDs	Levetiracetam	31 (4)	50 (3)	
	Lamotrigine	15 (2)	17 (1)	
	Other	15 (2)	33 (2)	
	None/Unknown	38 (5)	0	
Mother age (mean years ± SD)		41.8 ± 5.8	40.2 ± 8.9	
Education (median years)		16	16	
Father age (mean years ± SD)		42.3 ± 4.5	43.7 ± 9.6	
Education (median years)		16	16	
Children/family		1–3	2–4	
Family income (median range)		$105K–125K	$125K–$150K	

### Tantrum Calendar Reliability and Representativeness of Checklist Tantrums

Overall, 84% of check-in calls were completely consistent with subsequently submitted calendars. Inconsistencies were generally minor differences between telephone and calendar reported date, duration or intensity. One tantrum reported by phone was unnoted on the calendar.

For 44% of children, every calendar tantrum was also reported on the checklist. Overall, 69% of all calendar tantrums were reported on the checklist. There was no significant difference in mean duration of calendar and checklist tantrums (*t*_(21)_ = 1.02) reported for each child. Similarly, 86%–91% of overall severity ratings ranged from 2 to 4 for calendar and checklist tantrums, respectively; there was no difference in the severity distributions ($\chi_{\left(3\right)}^{2}$ = 0.54). Thus, tantrums reported by checklist were representative of a child’s tantrums overall.

### BRE vs. CPS Differences

Progressively greater percentages of sibs, CPS and BRE children had tantrums (Table 3); statistically, there was a trend for a higher percentage of children in the two epilepsy groups to have tantrums ($\chi_{\left(1\right)}^{2}$ = 3.19, *p* < 0.08). Table 3 also shows the BRE group had more frequent tantrums, the CPS group had the longest.

**Table 3:** Tantrum characteristics by group.

	Sibs	CPS	BRE
Percent children having tantrums	60	83	100
Percent tantrums triggered by child opposition	18	43	33
Frequency (/month-from calendar)	2.3 ± 3.0	3.7 ± 3.2	6.6 ± 6.6
Mean duration (min-from calendar)	2.1 ± 3.2	8.5 ± 10.6	2.8 ± 1.4
A/D Index (from post-tantrum report)	0.01 ± 0.55	−0.14 ± 0.44	0.2 ± 0.48
Aftermath: % Bad mood—Usual activity—Other	47–53−0	52–40−8	19–64−17

*A/D Index is the Anger Distress Index defined in “Materials and Methods” section*.

**Figure 1:**
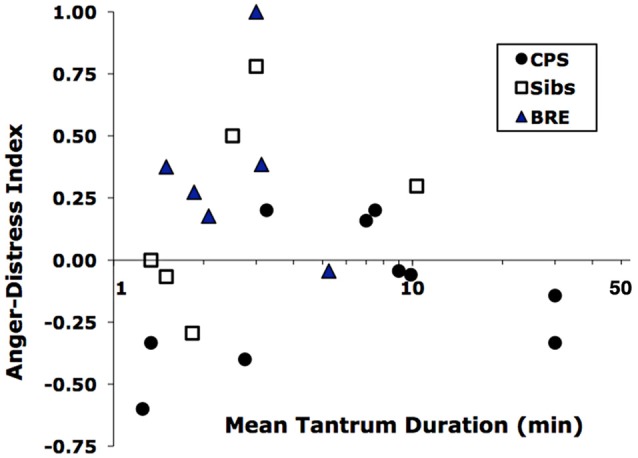
Distribution of tantrums of children in the Benign Rolandic epilepsy (BRE), complex partial seizures (CPS) and Sib groups by mean duration vs. Anger-Distress Index. Durations are log-scaled because of their wide range.

Generally, children with BRE experienced and/or expressed more anger than children with CPS who showed more sadness and distress. Thus, a MANCOVA with age as covariate showed an overall difference in tantrum frequency, duration and *A/D-I* among the three groups (Wilk’s Lambda = 0.45, *F*_(6,32)_ = 2.6, *p* < 0.05). Among the three variables, the *A/D-I* differed significantly across groups (*F*_(2,21)_ = 5.8, *p* < 0.02). Simple *post hoc* contrasts showed a significant difference between BRE vs. CPS in A/D-I (*p* < 0.01) and a trend for a difference in duration (*p* < 0.09). In fact, the combination of brevity and greater anger of BRE tantrums vs. longer, sadder CPS tantrums distinguished the two epilepsy groups, as shown by their nearly non-overlapping distributions on the duration-*A/D-I* plane (Figure 1). Additionally, considering the two most common post-tantrum categories, the CPS group were more likely to be in a “bad mood” than to “return to usual activities” while the BRE group was the reverse; siblings were intermediate (Table 3).

Trends in EEG emotion challenges were similar. A univariate ANOVA showed a trend for among-group differences in child-rated anger (*F*_(2,27)_ = 3.2, *p* < 0.06); a *post hoc* test showed higher BRE than CPS anger (*p* < 0.03). Siblings were intermediate. No such trends were found for child-rated fear or sadness (Parent and child ratings of the child’s emotion correlated significantly for two of the three challenges in each group: CPS: Fear *r* = 0.65, Anger = 0.59; BRE: Fear *r* = 0.52, Sadness *r* = 0.41). The videos showed that the majority of siblings smiled at least once during both Puzzle and Worst Prize episodes (55% and 78%, respectively), fewer BRE group children smiled (17% and 50%), and only one or two CPS group children smiled (10% and 17%). This difference in smiling was statistically significant for Worst Prize ($\chi_{\left(2\right)}^{2}$ = 7.88, *p* < 0.02).

Because the CBCL has no anger or sadness scales, *per se*, we compared the ratio of Aggression to Withdrawn scale scores across groups. They differed significantly. The BRE group had higher Aggression than Withdrawn scores on average (mean ratio = 1.1 ± 0.17) while the CPS and Sib groups had lower Aggression than Withdrawn scores (mean ratios = 0.96 ± 0.11 and 0.94 ± 0.09, respectively, *F*_(2,27)_ = 4.2, *p* < 0.05).

### Emotion/Behavior-EEG Correlations

#### Abnormality

**Figure 2:**
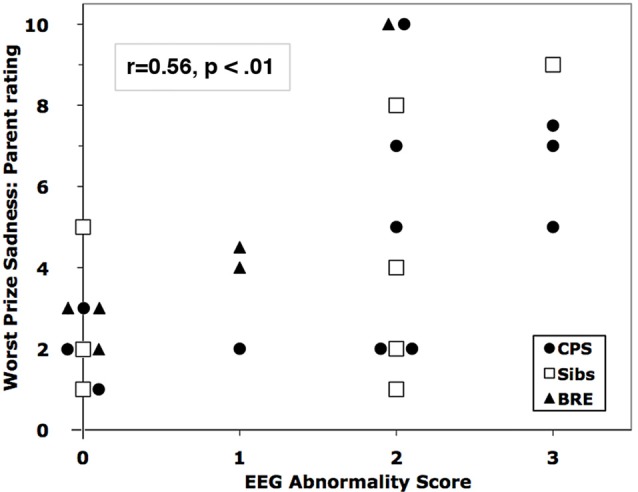
Correlation of parent-rated sadness in the Worst Prize challenge with EEG Abnormality Score. Overlapping markers have been slightly displaced to the left or right for clarity.

Four siblings of children with CPS had abnormal waveforms. Across all subjects, EEG Abnormality score correlated significantly with emotion ratings by one measure for each of the challenges: child self-rated fear for spider speech (*r* = 0.49) and parent ratings of observed anger for puzzles (*r* = 0.49) and sadness for the bad prize (*r* = 0.56, Figure 2). The three abnormal waveforms affected emotion responses differentially. Linear regressions showed that higher levels of IEDs and/or theta predicted significant increases in parent-rated anger (*F*_(3,27)_ = 4.24, *p* < 0.02) and sadness (*F*_(3,27)_ = 5.83, *p* < 0.005); delta had no significant effect (see Table 4 for the breakdown by abnormality).

**Table 4:** EEG abnormality effects on emotion: linear regression results.

	Parent rated anger (Puzzles)				Parent rated sadness (Worst prize)			
	**B ± SE**	**Beta**	*t*	*p*	**B ± SE**	**Beta**	*t*	*p*
Delta	−1.20 ± 0.95	−0.21	−1.26	0.22	0.50 ± 0.86	0.09	0.58	0.57
Theta	1.69 ± 0.43	0.52	3.05	0.005	2.01 ± 0.50	0.64	4.00	0.001
IED	2.00 ± 0.83	0.42	2.41	0.024	1.29 ± 0.75	0.28	1.72	0.10

*B, regression coefficient; Beta; standardized regression coefficient; SE, Standard Error*.

#### Laterality

**Figure 3:**
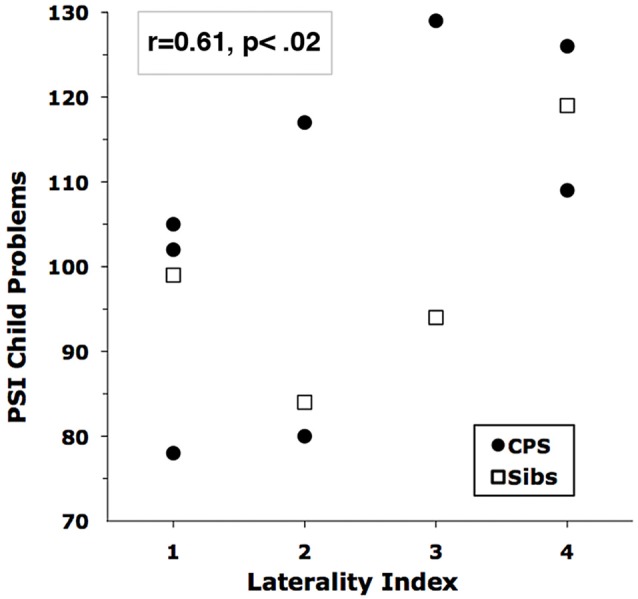
Correlation of Child Problems on the parenting stress index (PSI) with EEG Laterality Score. Higher Laterality Scores indicate more right-hemisphere involvement.

Across all subjects, the laterality of EEG abnormality was significantly, but oppositely, associated with Fear and Anger ratings. Fear correlated with left hemisphere localization (laterality index vs. child-rated speech fear, *r* = −0.69; vs. parent-rated fear, *r* = −0.47), but child-rated Anger correlated with right hemisphere localization (*r* = 0.56). The most striking correlation of right hemisphere localization was with the PSI scales for children with CPS. All but one of the PSI scales had *r*’s ≥ 0.36; the highest correlations were with Reinforce Parent (*r* = 0.69) and Acceptability (*r* = 0.58). Figure 3 shows the overall increase of Total Child Problems with right hemisphere localization. The addition of BRE group data reduced the PSI correlations, so these latter results may pertain only to children with CPS.

### Other Variables

Family demographics had no significant across-groups correlation with any emotion-response variables. Although several variables correlated moderately with younger age at first seizure, only two were statistically significant. Across all children with epilepsy, age at first seizure correlated with child-reported sadness, *r* = −0.54, *p* < 0.05. For children with CPS, age at first seizure correlated with tantrum frequency, *r* = −0.69, *p* < 0.01. AED status of the two epilepsy groups is shown in Table 2. Two thirds of children with CPS and all of those with BRE were on AEDs at time of testing. Of the AED-treated children, all but two were on monotherapy. There were no differences among AED subgroups in Table 2 on any variable reported here.

## Discussion

Associations between pediatric epilepsy and children’s E/B problems are well established. Some major risk factors are known, but why some children experience internalizing problems, others display externalizing problems, while still others have only average problems remains unclear. Our direct observations of emotion responses in the laboratory and parent-reported tantrums at home clarify which children are likely to be affected and what problems their EEG might predict. Results suggest that children with BRE react with more anger and aggression; those with CPS react with more sadness and withdrawal. Across both groups, left hemisphere localization of abnormal EEG waveforms was associated with greater fear of public speaking, while right hemisphere localization was associated with greater anger. Right hemisphere localization was also associated with parent-reported problems at home in children with CPS. Increased EEG waveform abnormality correlated with heightened negative emotions in all groups.

The trend for increased tantrums in our sample is consistent with older reports (Keating, 1961; Nuffield, 1961; Elithorn, 1974). The 60% prevalence of tantrums among siblings is also higher than 25%–50% estimates reported in the literature. A higher proportion of sibling tantrums were reportedly triggered by squabbles with siblings (29% vs. 4%–7% for the epilepsy groups). This may indicate that having a sibling with epilepsy increases conflict among children in the family.

Children with BRE tend to have more frequent, but briefer, angry tantrums and then get over it; children with CPS have longer, sadder tantrums and are more likely to be in a bad mood afterward. Similarly, children with BRE showed more anger in response to challenge while children with CPS were least likely to smile. That BRE is associated with more intense anger while CPS associates with stronger distress supports previous findings of externalizing and internalizing problems in BRE vs. CPS, respectively (Weglage et al., 1997; Croona et al., 1999; Yung et al., 2000; Massa et al., 2001, vs. Schoenfeld et al., 1999; Caplan et al., 2004).

The localization and degree of abnormality of EEG waveforms may now help explain why only some children with epilepsy have E/B problems. Our correlational analysis confirmed previous comparisons between epilepsy and control groups (Mathiak et al., 2009) in that children’s abnormal right hemisphere waveforms are associated with greater anger, behavioral difficulties and conflict with parents (Note that PSI items are phrased in terms of complaints about child misbehavior and therefore maximize parent report of such problems).

Across diagnostic groups, greater interictal waveform abnormalities were associated with more intense emotion responses. These results resemble previous subgroup differences (Massa et al., 2001), and also highlight EEG waveforms that may predict E/B problems. Theta activity and spikes predicted emotion reactions, but delta did not, which is consistent with observations that delta range slowing is a more generalized phenomenon that may be found in individuals without seizures (Reiher et al., 1989; Hughes and John, 1999). Use of different AEDs within a study can complicate interpretation. Here, we saw no E/B differences among children treated with levetiracetam, other AEDs or no AEDs. In any event, our correlations are between behavior and on or off-medication EEG, i.e., brain activity as it was measured, however it came to be that way. Thus, the important but separate issue of how AEDs may affect behavior is irrelevant to the results as presented.

The systematic relationship between neurological effects, represented by abnormal waveforms, and E/B function highlighted here contrasts with findings that life stress (low socio-economic status, poor parental health) contributes to mental health problems associated with pediatric epilepsy (Ott et al., 2003; Baum et al., 2010). However, our participating families were relatively high SES and reported few stressors. These demographics, together with the lack of family SES effects on emotion variables and the use of sibling controls suggests that environmental stress is probably limited in this study and neurological factors more likely to be causal. As has been noted, the effect of adverse biological factors becomes clearer when environments are benign (Raine, 2002).

Epilepsy-related pathophysiology may shift the neural circuitry of emotions in one or another direction. Our EEG findings suggest that emotion changes in epilepsy fall along two dimensions, laterality and abnormality. Lateralization shifts the balance between anxiety and anger, with left foci associated with excessive anxiety and right foci with excessive anger. If seizure foci reduce ipsilateral interictal function (via, e.g., hypometabolism, Benedek et al., 2006; Badawy et al., 2009), thereby disinhibiting the contralateral hemisphere (Reggia et al., 2001), our results would be entirely consistent with the standard model of left hemisphere activating anger and right hemisphere activating anxiety (Thibodeau et al., 2006; Harmon-Jones et al., 2010).

Along the abnormality dimension, intensity of all negative emotions may rise with increasingly abnormal EEG waveforms. The general tendency to experience negative emotions more frequently and intensely has been called “Negative affectivity”, and is a major childhood precursor to clinical anxiety and depression (Lonigan and Vasey, 2009). Thus, epilepsy-related brain pathophysiology, indicated by abnormal EEG theta and IEDs, may increase negative affectivity, leading to clinical psychopathology. Indeed, animal models show that the “kindling” of limbic seizures increases interictal fearfulness (Depaulis et al., 1997; Adamec, 2003). Finally, the respective shifts toward anger vs. anxiety in BRE vs. CPS emotion reactions suggests that epilepsy type determines which and/or how neural circuits are affected.

Practically, the direct and possibly causal connection between epilepsy and E/B problems suggested here implies that attention to behavioral issues and referral for appropriate psychotherapy and behavior management training for parents should be a routine part of clinical care for children with epilepsy. Because behavior related abnormal waveforms detected in an office visit time-frame appear to predict E/B problems, clinical EEGs might enable physicians to anticipate what problems a child is likely to have and explain this to parents. Lastly, while some AEDs adversely affect behavior (Ronen et al., 2000), lamotrigine and oxcarbazepine are already used as mood stabilizers in children (Reijs et al., 2004). Our findings provide additional rationale for assessing treatment of epilepsy-related E/B problems with select AEDs (Pressler et al., 2005).

Our scoring of abnormal EEG waveforms is novel and requires replication. The stability of the hemispheric localizations is also unknown. The frequency of IED “migration” in children, leading to “false” localizations, has long been debated (Andermann and Oguni, 1990 vs. Blume, 1990). Reports of interhemispheric migration range from never (Blume and Kaibara, 1991) through 0.1% (Lundervold and Skatvedt, 1956) to 10% (Lee et al., 2010) and 20% of foci (calculated from Table 1 and Figure 3 in Konishi et al., 1994). Whether apparent migration results from seizure propagation, multifocality or other processes, future studies should determine which, if any, foci migrate and whether this is associated with any behavioral change. However, the chief limitation of this study is modest sample sizes, as acknowledged by the phrase “Exploratory observations…” in our title. Some effects do replicate earlier work (more severe problems correlate with earlier age of seizure onset) and significant differences were found with small numbers of subjects, suggesting consistency with the literature and robustness of results. Nonetheless, our conclusions must remain working hypotheses until experimental effects are replicated.

## Author Contributions

MP designed the study, analyzed the data and wrote the manuscript. EHD added the public speaking component to the Spiders episode and supervised testing. JTM reviewed the EEG literature, designed the recording protocol and analyzed the EEG data. EHD and JTM edited the manuscript.

## Disclosure

We confirm that we have read the Journal’s position on issues involved in ethical publication and affirm that this report is consistent with those guidelines.

## Conflict of Interest Statement

The authors declare that the research was conducted in the absence of any commercial or financial relationships that could be construed as a potential conflict of interest.
